# Machine Learning Assessment of Spasmodic Dysphonia Based on Acoustical and Perceptual Parameters

**DOI:** 10.3390/bioengineering10040426

**Published:** 2023-03-28

**Authors:** Federico Calà, Lorenzo Frassineti, Claudia Manfredi, Philippe Dejonckere, Federica Messina, Sergio Barbieri, Lorenzo Pignataro, Giovanna Cantarella

**Affiliations:** 1 Department of Information Engineering, Università degli Studi di Firenze, 50139 Firenze, Italy; 2 Genetics, Oncology and Clinical Medicine, Università degli Studi di Siena, 53100 Siena, Italy; 3 Federal Agency for Occupational Risks, 1020 Brussels, Belgium; 4 Department of Clinical Sciences and Community Health, University of Milan, 20122 Milan, Italy; 5 SC Neurofisiopatologia, Fondazione IRCCS Ca’ Granda Ospedale Maggiore Policlinico, 20122 Milan, Italy; 6 Department of Otolaryngology, Fondazione IRCCS Ca’ Granda Ospedale Maggiore Policlinico, 20122 Milan, Italy

**Keywords:** spasmodic dysphonia, voice assessment, acoustical analysis, BioVoice, machine learning, LIME

## Abstract

Adductor spasmodic dysphonia is a type of adult-onset focal dystonia characterized by involuntary spasms of laryngeal muscles. This paper applied machine learning techniques for the severity assessment of spasmodic dysphonia. To this aim, 7 perceptual indices and 48 acoustical parameters were estimated from the Italian word /a’jwɔle/ emitted by 28 female patients, manually segmented from a standardized sentence and used as features in two classification experiments. Subjects were divided into three severity classes (mild, moderate, severe) on the basis of the G (grade) score of the GRB scale. The first aim was that of finding relationships between perceptual and objective measures with the Local Interpretable Model-Agnostic Explanations method. Then, the development of a diagnostic tool for adductor spasmodic dysphonia severity assessment was investigated. Reliable relationships between G; R (Roughness); B (Breathiness); Spasmodicity; and the acoustical parameters: voiced percentage, F2 median, and F1 median were found. After data scaling, Bayesian hyperparameter optimization, and leave-one-out cross-validation, a k-nearest neighbors model provided 89% accuracy in distinguishing patients among the three severity classes. The proposed methods highlighted the best acoustical parameters that could be used jointly with GRB indices to support the perceptual evaluation of spasmodic dysphonia and provide a tool to help severity assessment of spasmodic dysphonia.

## 1. Introduction

Dystonia refers to a set of movement disorders typically characterized by involuntary sustained or intermittent muscle contractions that may affect anatomical areas, such as neck muscles (cervical dystonia); the orbicularis oculi muscles (blepharospasm); or laryngeal muscles, causing laryngeal dystonia [1]. This latter condition is characterized by a relatively low prevalence, 1 ÷ 100,000 [2]. Two main different types of laryngeal dystonia have been described: adductor spasmodic dysphonia (AdSD) and abductor spasmodic dysphonia (AbSD), which rarely occur together [1,3]. AdSD represents an impairing disease that affects adductor muscles of the vocal folds during phonation, causing irregular voice breaks and a typically strained, strangled voice; it is the most common type of spasmodic dysphonia (SD), affecting 90% of cases [2], with a female predominance and an average age of onset at 45 years [2]. Spasms tend to occur during vowel emissions, mainly during the glottal stop between them [1]. AbSD concerns vocal fold abductor muscles, causing glottis opening during voice production and consequent breathy vocal breaks, especially when a voiceless consonant comes before a vowel [1]. Patients commonly perceive high voice fatigue due to the respiratory effort required to produce an intelligible word or sentence.

SD can have a devastating negative impact on verbal communication and on social and professional relationships. Indeed, in the most severe cases, speech fluency is highly reduced, and intelligibility is strongly compromised. SD etiopathogenesis remains unclear: early studies assumed psychosomatic causes, but it was later found out that patients diagnosed with SD showed central nervous system anomalies [2]. In more recent studies, thanks to neuroimaging techniques such as PET and fMRI, SD was associated with disorders of the basal ganglia and cerebellum [2,4]. The review by Hintze et al. [2] highlighted three possible neurological mechanisms: reduced cortical inhibition, sensory processing disturbances, and neuromorphological alterations.

At present, the diagnostic assessment of SD is mainly based on clinical investigation and on perceptual voice evaluation, but some methods based on acoustical and aerodynamical parameters have been proposed to define voice characteristics of AdSD patients, mainly for pre-post treatment comparison [5,6,7]. Such methods can be used in the clinical assessment of SD; however, perceptual indices present limitations due to subjectivity and possible different levels of experience of the examiners [8].

The lack of strict scientific criteria and objective means makes SD diagnosis difficult and leads to diagnostic delays: a study [9] evaluated a delay of about 4.43 years before the correct diagnosis, due to similar clinical features such as muscle tension dysphonia (MTD), at least in its hyperkinetic form, and essential tremor. Indeed, this latter disorder, characterized by more regular oscillations of multiple laryngeal muscles, is not easily distinguished from SD because its rhythmic patterns can be related to apparently regular voice breakings caused by SD. Similarly, MTD presents multiple laryngeal muscle contractions, leading to a strained voice. Nevertheless, significant differences were observed for AdSD but not for MTD between sustained vowels and connected speech voice samples through the Cepstral Spectral Index of Dysphonia assessment [8], supporting the assumption of MTD being task independent. These results suggested that particular voice features obtained with objective acoustical analysis may represent relevant biomarkers in SD assessment. Moreover, machine learning strategies have been widely implemented in the last decades to automatize these tasks. As far as the distinction between healthy subjects (HS) and SD patients is concerned, Schlotthauer et al. [10] obtained 93% accuracy applying an artificial neural network and support vector machine techniques (SVM) to the sustained vowel /a/. A similar result with 95% accuracy was achieved by Costantini et al. [11] with Naïve Bayes, SVM, and Multilayer Perceptron models using features extracted from the sustained vowel /e/. Powell et al. [12] managed to distinguish seven voice pathologies from HS, including SD, with 55% overall accuracy. This result was slightly improved by Hu et al. [13], who obtained 67% accuracy when considering four diseases and HS. In Fang et al. [14], a deep neural network was applied to eight vocal illnesses and HS with an overall accuracy of 90% for female subjects. In this latter paper, however, no distinction was made between AdSD and vocal tremor as they were generically labelled as laryngeal dystonia. Finally, acoustical analysis and machine learning have been used also to assess the effectiveness of SD treatments: Suppa et al. [7] highlighted that botulinum neurotoxin type A injection improved voice quality, although it was not completely restored, while Prudente et al. [4] proved that repeated transcranial magnetic stimulation provided significant improvements to four acoustical parameters. However, to the authors’ knowledge, no study focused on the analysis of both perceptual and objective parameters to assess severity of AdSD, and at present, little attention has been paid to possible relationships between them.

The purpose of this work was to investigate the implementation of acoustical analysis combined with machine learning algorithms to develop a non-invasive system characterizing the severity of AdSD. Specifically, it aimed at finding possible relationships between objective parameters and perceptual indices and evaluating AdSD severity through machine learning voice assessment (MLVA) techniques. This approach could support speech pathologists and otolaryngologists in grading AdSD severity and in monitoring its evolution over time and treatment [15].

## 2. Material and Methods

Voice samples were collected from 28 female patients (mean = 64.6 years, std = 11.6 years) diagnosed with AdSD and recruited at Ospedale Maggiore Policlinico Milano, Milano, Italy. Four male patients were recorded as well, but they were excluded from further analyses to avoid unbalanced data issues.

Each patient underwent a complete phoniatric investigation including videolaryngostroboscopy. The diagnosis was unanimously made by a team consisting of a highly experienced laryngologist and two voice therapists, both with a long period of experience with SD patients. Several patients were known to suffer from SD for years and had already been successfully treated with botulinum toxin.

All subjects uttered only once, at comfortable pitch and loudness, an Italian standardized sentence rich in vocalic sounds and constantly voiced: “il bambino ama le aiuole della mamma” (“the child loves mother’s flowerbeds”). Recordings were collected anonymously with smartphone-integrated microphones in a controlled (quiet) room. This study was approved by the Institutional Review Board of the Fondazione IRCCS Ca’ Granda Ospedale Maggiore Policlinico, Milano, Italy, and informed consent was obtained from each participant.

### 2.1. Perceptual Parameters

Perceptual evaluation of the whole sentence was performed blindly by three independent judges with experience in voice disorders (1 otolaryngologist and 2 voice therapists), and scores were averaged. The scores ranged from 0 to 10, where 0 is the rating for the worst voice condition and 10 for the best one. Seven perceptual parameters were considered. Three of them were taken from the GRB scale proposed by Hirano [16] for voice assessment:

- G (global grade of dysphonia): the judgement is based on the overall impression of voice quality deterioration;
- R (Roughness): the impression of irregular F0 and of noise;
- B (Breathiness): turbulent noise related to air escape through the vocal folds.

The GIRBAS scale, derived from [16], represents the most used protocol for voice assessment and is currently suggested for clinical research by the European Research Group [17] and the Società Italiana di Fonologia e Laringologia [18]. Indices I, A, and S were not included in this work as they are less reliable in clinical settings [19].

Other four perceptual parameters were taken from the IINFVo scale that was defined for substitution voicing assessment by Moerman et al. [20] and later validated by Siemons-Luhring [21] for SD assessment as well:

- I (Intelligibility): the impression of the possibility of the patient to be understood by the listener.
- F (Fluency): intended as smoothness of speech production.
- Vo (Voicing): the capability to correctly utter voiced or unvoiced speech, that is, the speech is voiced or unvoiced when it actually needs to be voiced or unvoiced [20].
- S (Spasmodicity): this parameter is related to the perception of voice breaks, tremor, and strain.

### 2.2. Acoustical Analysis

The Italian word /a’jwɔle/ was manually segmented from the standardized sentence and analyzed with the BioVoice open-source software tool [22,23], already successfully applied to the acoustical analysis of voices from newborns to children and adults, such as shape classification of newborn cry melody [24], dysprosody detection in Parkinson’s disease [25], and genetic syndrome speech phenotype characterization [26]. The word /a’jwɔle/ is widely used as it is mainly composed of vowels and is also suited for studying articulation capabilities. BioVoice automatically resamples audio files at 44.1 kHz, saves them in .wav format, and sets proper frequency ranges for male/female/child/newborn voices to obtain reliable acoustical parameters, both in the time and in the frequency domain. Specifically: frame duration, number, and percentage of voiced/unvoiced parts, F0, formants F1–F3, jitter, and noise (Normalized Noise Energy, NNE), along with their standard deviation, are estimated. BioVoice also computes the Power Spectral Density (PSD), normalized to its maximum value. In this work, the PSD frequency spectrum in the adult’s range up to 5500 Hz was divided into 500 Hz wide slides where the average power was calculated [27]. A total of 48 acoustical parameters were estimated, summarized in Table 1, along with a short description. Other parameters are specific for newborn/infant cry and for the singing voice. As they were not used in the present work, they are not listed here.

### 2.3. Machine Learning and Statistical Analysis

Automatic assessment of voice pathologies based on artificial intelligence techniques represents an established approach to analyze high-dimensional data. Most of the used models include k-nearest neighbors (KNN), support vector machine (SVM), and random forest (RF), and they were implemented in this work. Some advantages of machine learning (ML) methods are that very few assumptions are required about the data-generating systems, which are instead relevant to obtain reliable statistical results but are not often available in clinical practice. Moreover, they are capable of generalizing underlying data patterns for prediction with completely new observations [30,31,32]. Furthermore, many studies [11,33] have highlighted that even when data do not show statistically significant differences, ML can still effectively identify parameters to differentiate observations. As the present study is focused on prediction rather than inferencing, statistical analysis was implemented only to support ML results. The items listed in Table 1 were used as features for a set of multiclass and multivariate classification experiments. Features were z-score normalized to allow comparisons between variables with different scales and units of measurement. They made up the whole training set. Hyperparameter tuning was performed with Bayesian optimization using MATLAB^®^ 2020b [34]. The function *bayesopt.m* allows for the selection of several properties, such as the number of iterations for the evaluation of the objective function (set at 60 iterations, according to [35]); the optimization metric, which corresponds to the global accuracy; and model hyperparameters that minimize the overall misclassification error. In our study,

- For the KNN classifier: the number of neighbors k was evaluated between 2 and 27. The considered distance metrics were “cityblock”, “Chebyshev”, “correlation”, “cosine”, “Euclidean”, “hamming”, “jaccard”, “mahalanobis”, “minkowski”, “seuclidean”, “spearman” (according to [36]). The distance weight was chosen between “equal”, “inverse”, “squared inverse”.
- For the SVM classifier: coding was selected between “one vs. one” or “one vs. all”. Box constraint and kernel scale were evaluated between 10^−3^ and 10^3^. The kernel function was set as Gaussian.
- For random forest, the *fitcensemble.m* function was used, and the aggregation method was set as “Bag”. The minimum number of leaves was selected between 2 and 27. The maximum number of splits was between 2 and 27. The split criteria were between “deviance”, “gdi”, and “twoing” (according to [37]). The number of variables to sample was between 1 and 55.

To reduce overfitting and obtain reliable results, the trained models were cross-validated with the leave-one-subject-out (LOSO) method. Perceptual indices were used to separate AdSD severity into three classes: severe, moderate, and mild, as shown in Table 2.

In the first artificial intelligence (AI) experiment, each rating from the GRB and the IFVoS scales was iteratively implemented as a response variable to identify relationships between objective and perceptual variables and find the most relevant features that define the voice characteristics of AdSD. At each iteration, only one perceptual parameter was considered, and the other ones were ignored.

In the second AI experiment, index G was used as response variable, while all other parameters were used to evaluate the classifiers capability in AdSD severity assessment. In Table 3, the number of patients for each class is shown. Categorical values were not z-score normalized. A MATLAB^®^ 2020b [34] code was developed to automatically save validation accuracies, precision, sensitivity, specificity, F-score (i.e., the harmonic mean between precision and sensitivity), area-under-the-curve values (AUCs), and model hyperparameters.

An important issue concerning machine learning techniques, especially when they are used as diagnostic tools for decision making, is the trust of individual prediction: although convincing, it can be difficult to directly relate prediction results to existing physiological knowledge [32]. Evaluation metrics such as accuracy may not be indicative; therefore, Ribeiro et al. [38] proposed textual and visual artefacts that provide a qualitative explanation of the relationships between single observation (or instance) features and model predictions. Thus, Local Interpretable Model-Agnostic Explanations (LIME) were used to identify which parameters contributed or disagreed with the prediction of an individual observation made by the trained models. The LIME method simplifies the relationship between data labels and independent variables and is based on the assumption that every model behaves like simple linear models at the local scale, i.e., at single-row-level data. Once a particular instance is selected, or “explained”, LIME operates in three steps:

(1) It samples new data from the instance and calculates distances between sampled data and the original observation.
(2) It uses the complex model that needs to be explained to make predictions on synthesized data and then it trains the simple model.
(3) The simple model is weighted using the same distance metric of step 1 and therefore it identifies the features that contributed in order to obtain a specific prediction.

**Figure 1:**
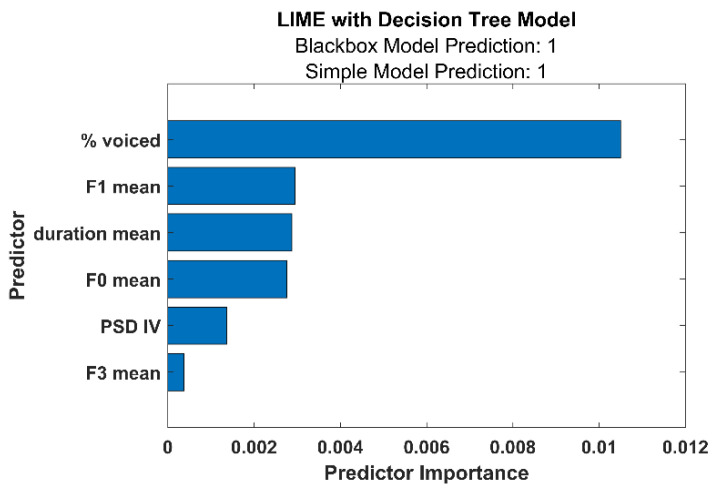
LIME MATLAB output: weights of the acoustical parameters for a specific observation.

In MATLAB, the function *lime.m* requires the user to specify the hyperparameters of the trained complex model, the observation that needs to be explained (named “query point”), and the number of predictors to train the simple model, and it returns a bar graph showing the relevance of each predictor and predictor weight. A code was developed to iteratively compute LIME for all observation for each trained classifier, setting the number of features to 10. As an example, Figure 1 depicts the outcome of LIME prediction for a row of the dataset; hence, the query point or observation of the first AI experiment, considering G as response variable. Blackbox model refers to the trained complex classifier, Simple Model to the classifier trained by LIME, and 1 to the severity class introduced in Table 2. On the vertical axis, features that contributed to the correct classification are shown (in this case: % voiced, F1 mean, duration mean, and F0 mean), while in the horizontal axis, their individual weights are shown.

With reference to Figure 1, in order to find relevant features, other models and corresponding plots were obtained for each observation, and predictor weights were averaged. Then, the total weight was computed, and the ratio between individual predictor weight and total weight was considered in order to identify relevant parameters: an arbitrary threshold was set at 0.1.

As for statistical analysis, a Shapiro–Wilk test proved that data were normally distributed in both experiments. Therefore, in order to trace AI experiment settings, a first ANOVA test was performed, taking perceptual indices as response variables and acoustical parameters as dependent variables.

A second ANOVA test was carried out, considering G as a response variable and all other parameters as dependent variables. Significance level was set at 0.05 for both tests.

## 3. Results

Table 4 summarizes the best validation accuracies of the first experiment, in which perceptual indices were iteratively used as response variables to highlight a possible relationship between them and objective parameters. Table 4 displays models’ hyperparameters as well: d stands for distance metric, w for distance weight, c for split criterion, v for number of predictors to select at random for each split, l for number of leaves, s for the maximum number of splits, and N for the number of learning cycles.

LIME trained a simple model for each observation, weighted the simple model with the classifiers listed in Table 5, and computed which features contributed to the outcome; therefore, it was possible to find out relationships between response variables and acoustical correlates, as Table 5 shows.

**Figure 2:**
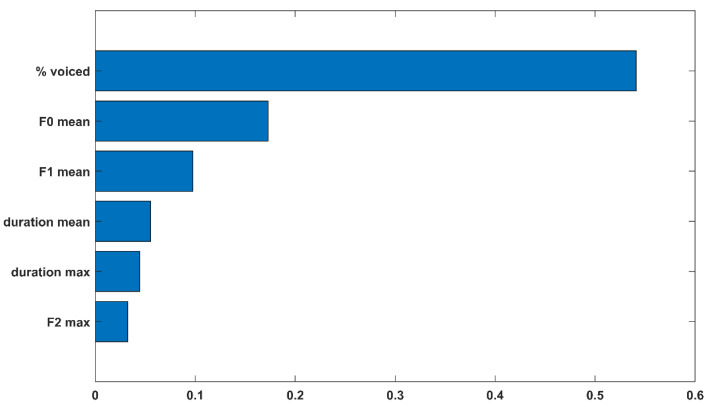
Acoustical parameters sorted in descending order on the basis of the ratio between their weight and the total weight for the G index.

**Figure 3:**
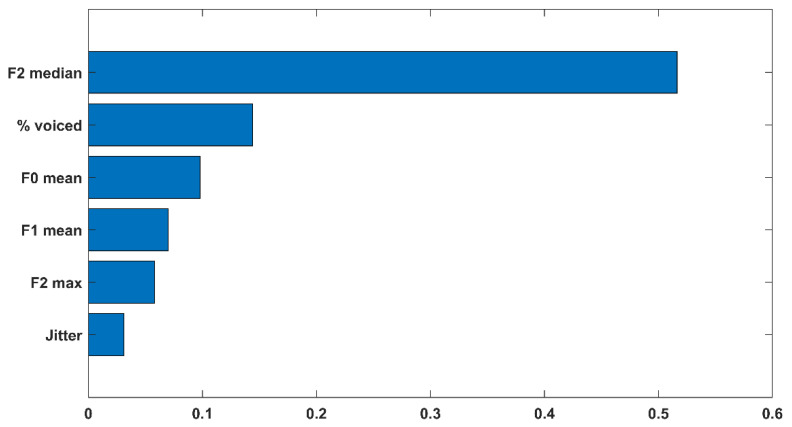
Acoustical parameters sorted in descending order on the basis of the ratio between their weight and the total weight for the R index.

**Figure 4:**
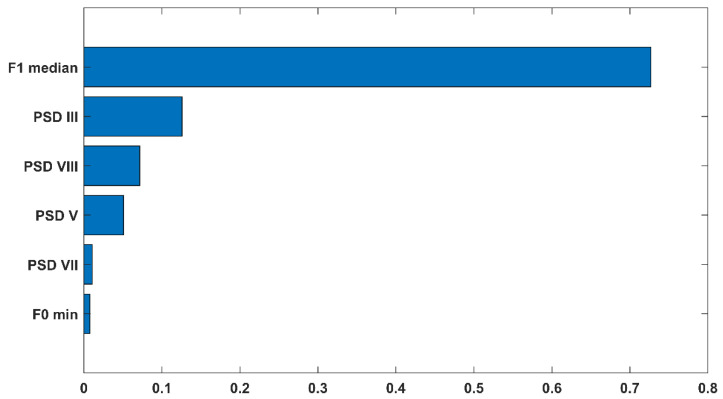
Acoustical parameters sorted in descending order on the basis of the ratio between their weight and the total weight for the B index.

**Figure 5:**
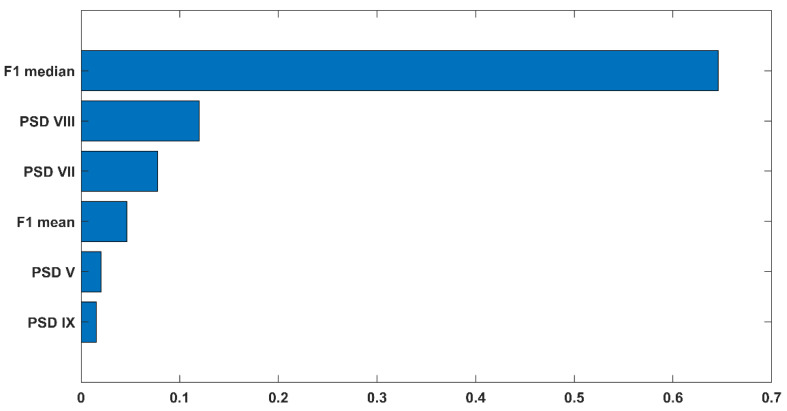
Acoustical parameters sorted in descending order on the basis of the ratio between their weight and the total weight for the Spasmodicity index.

Figure 2, Figure 3, Figure 4 and Figure 5 show the percentage of total weight explained by each feature, respectively for G, R, B, and Spasmodicity.

In the second experiment, G was used as response variable to assess AdSD severity with both perceptual and objective parameters. The best model was a KNN with k = 2 and Euclidean metric distance, which reached 89% LOSO validation accuracy. LIME results are summarized in Table 6.

In Table 7, the performance of the KNN model is presented. In brackets, the 95% confidence bounds for each metric are reported.

In Table 8, results from the first ANOVA test are shown with *p*-values in brackets. No significant difference between classes for the B index was observed.

For the second statistical test, perceptual indices showed significant differences between severity classes for R (*p* < 0.001), B (*p* = 0.029), Intelligibility (*p* < 0.001), Fluency (*p* < 0.001), Voicing (*p* < 0.001), and Spasmodicity (*p* < 0.001). Furthermore, G showed significant differences between classes for F0 mean (*p* = 0.012), F0 median (*p* = 0.043), and F0 max (*p* = 0.003).

## 4. Discussion

The identification of relationships between perceptual and objective measures of voice still represents a topic under discussion. In Dejonckere et al. [39], correlations were found between G, shimmer, and HNR; R and jitter; and B and shimmer. In another paper, the same authors showed that G was also highly correlated (0.71) with the dominant cepstral peak [40]. Bhuta et al. [17] found relationships between the voice turbulence index (VTI) and G, NHR with G and R, and soft phonation index (SPI) with G and B. These results were confirmed in more recent papers of Park et al. [41] and Narisimhan et al. [42]. Focusing on SD, Dejonckere et al. [43] found relationships only between B and devoicing measures extracted with AMPEX: PVF (i.e., the proportion of voiced frames), PVS (i.e., the proportion of speech frames), and VL90 (which represents the 90th percentile of the voicing length distribution). However, this study had different aims from the present one, as it concerned the comparison between pre- and post-treatment voice quality.

In the present study, the LIME method was implemented to investigate relationships between perceptual indices and objective parameters for AdSD, which represent an unexplored task. Specifically,

- The percentage of voiced parts in the whole audio signal (% voiced) was the parameter most strongly related to G value. This may be linked to repeated interruptions that significantly lower voice quality. This result is supported also by the two parameters: duration mean and duration max, which highlight the longer time required to emit the word /a’jwɔle/ due to alterations in vocal fold mobility. F0 mean and F1 mean contributed as well. The relevance of F1 mean could be associated with pharynx constriction degree [44].
- R assessment is linked to F2 median and % voiced. These results suggest that roughness is related to tongue movements [44]. Jitter was found to be relevant in 8 out 28 predictions with LIME, but its total contribution weight was equal to 3.1%. Interestingly, jitter represented a “confounding” parameter causing moderate cases of AdSD to be classified as severe. In 25% of observations, NNE, a measure of noise alternative to HNR, was considered relevant, but its total contribution weight was equal to 2.4% only. However, it is important to specify that these discrepancies with literature results [39,41,42] are probably caused by different tasks: while jitter and NNE typically show strong correlations with R when healthy subjects are compared with patients diagnosed with AdSD, probably jitter and NNE do not represent relevant parameters to assess and distinguish among different AdSD severity classes.
- B ratings and F1 median values showed the strongest relationship. As shown in Figure 4, even if PSD III (range [1–1.5] kHz) is considered a relevant parameter, it caused two out of four misclassifications from moderate to mild, and therefore it should be discarded.
- Spasmodicity is associated with F1 median as well as the medium–high frequency region of the spectrum, described by PSD VIII (range of 3.5–4 kHz).

For Intelligibility, Fluency, and Voicing, the low validation accuracies of trained classifiers made the relevant parameters identified by LIME unreliable. However, statistical analysis highlighted significant differences between classes for Intelligibility in Pause duration min (0.049), which can be related to high correlation between Intelligibility and PVF in [21], and F0 mean (0.041). Fluency presented significant differences in NNE (*p* = 0.037) and PSD I (*p* = 0.01), with this last parameter confirmed by LIME as well, and Voicing with F2 min (*p* = 0.03) and F0 mean (*p* = 0.005), in agreement with Table 5.

It is interesting to notice that the LIME outcome is supported by statistical analysis, especially for R: indeed, F2 median, % voiced, and F1 mean show significant differences between classes, in particular between severe and moderate class with *p* = 0.005, *p* = 0.040, and *p* = 0.040, respectively. This is partly true also for G and Spasmodicity, where F0 mean and F1 median each showed significant differences. For Spasmodicity, the ANOVA highlighted that, besides F1- and F2-related measures, % voiced, Number of Units, and Number of pauses provided significant differences between severity classes: since spasmodicity directly depends on voice breakings, such results reflect strong connection with objective correlates.

Both acoustical and perceptual parameters were included as features in a multiclass classification experiment that aims at developing a model capable of supporting otolaryngologists in AdSD assessment. To the authors’ knowledge, this represents the first attempt to assess the severity of such pathology with machine learning techniques. Indeed, the literature mainly concerns general voice disorder, or just SD assessment, or only the discrimination between SD and MDT.

With a KNN model, a high accuracy of 89% was obtained: this is a promising result that highlights how MLVA could be successfully applied as a support for ENT specialists in AdSD assessment. Table 7 shows high precision, F-score, and AUC values, especially for the severity class 1, which means that, with this classifier, acoustical parameters and perceptual indices are able to distinguish the most severe cases of AdSD. Our model also shows strong recognition capabilities for moderate cases of AdSD, while mild severity cases are characterized by lower performance. In fact, our sample comprised only two mild cases. Hence, mild cases of SD were underrepresented in our study, and further analyses will be required in the future; this is possibly a consequence of our severe inclusion criterion, i.e., only cases with compelling clinical diagnosis of SD. However, precision and specificity are equal to 100%, underlying that no severe or moderate cases were misclassified as mild cases: this is an important result, useful to avoid underestimation. LIME was also used to identify relevant features in prediction: Table 6 shows that they were all perceptual parameters, particularly Spasmodicity, R, and Voicing. Moreover, ANOVA detected significant differences between all perceptual parameters (especially R, Voicing, and Spasmodicity, with *p*-values < 0.001) and F0-related measures, which can be associated with PSD I. These results confirm the validity of the IINFVo scale for SD assessment [21].

For each perceptual index, the proposed method was capable of detecting the best corresponding acoustical feature: % voiced for G, F2 median for R, and F1 median for B and Spasmodicity. These parameters could be associated with voice breakings caused by spasms, tongue movement limitations, and pharynx constriction, respectively, which may also depend on anomalous laryngeal muscle contraction of AdSD. Due to the low validation accuracy of the trained models, possible relationships between IINFVo and acoustical parameters were not considered, and further analysis is required to improve classification performances. However, the combination of machine learning techniques, which can highlight underlying patterns in data, and LIME, allowed for the identification of useful relationships between perceptual and acoustical parameters in SD assessment, a task where statistical analysis methods showed poor performances. Finally, a KNN model provided 89% overall accuracy in dividing patients affected by AdSD into three severity classes—this promising result could help ENT specialists to recognize subjects that would benefit from botulinum toxin A treatment and reduce pathology gravity underestimation.

## 5. Conclusions

AdSD is characterized by strained voice, pitch breaks, and intermittent breathiness that are usually assessed with perceptual ratings. Acoustical analysis and machine learning techniques can support the clinicians’ diagnosis providing reliable relationships between objective and perceptual indices. In this paper, an innovative approach is proposed to address this problem on the basis of the LIME method, which highlighted the most relevant objective parameters that should be taken into account to support and validate G, R, B, and Spasmodicity assessment. This could be helpful to quantify possible improvements or worsening of voice quality after specific treatments. Results were supported by statistical analysis as well. Moreover, a novel machine learning experiment was carried out to develop an automatic tool that could help otolaryngologists in AdSD severity assessment and also to monitor SD voice quality over time after botulinum toxin injection, as an aid to check whether and to what extent it was successful and if and when it may be appropriate to repeat the treatment. However, adjustments of the method and a larger dataset, including male patients as well, for validation are required to reduce the number of severe cases classified as moderate.

## Figures and Tables

**Table 1:** Acoustical analysis of /a’jwɔle/: BioVoice parameters.

Parameter	Description
**F0 mean [Hz]** **F0 median [Hz]** **F0 std [Hz]** **F0 min [Hz]** **T0(F0 min) [s]** **F0 max [Hz]** **T0(F0 max) [Hz]** **Jitter (local) [%]** **NNE** F* **mean [Hz]** F* **median [Hz]** F* **std [Hz]** F* **min [Hz]** F* **max [Hz]** **Signal duration [s]** **% voiced** **Voiced duration [s]** **Number of units** **Duration mean [s]** **Duration std [s]** **Duration min [s]** **Duration max [s]** **Number pauses** **Pause duration mean [s]** **Pause duration std [s]** **Pause duration min [s]** **Pause duration max [s]** **PSD I [dB]** **PSD II [dB]** **PSD III [dB]** **PSD IV [dB]** **PSD V [dB]** **PSD VI [dB]** **PSD VII [dB]** **PSD VIII [dB]** **PSD IX [dB]** **PSD X [dB]** **PSD XI [dB]**	Mean of fundamental frequency Median of fundamental frequency Standard deviation of fundamental frequency Minimum of fundamental frequency Time instance at which the minimum of F0 occurs Maximum of fundamental frequency Time instance at which the maximum of F0 occurs Frequency variations of glottis cycle-to-cycle periods [28] Normalized noise energy [29] Mean of * formant Median of * formant Standard deviation of * formant Minimum of * formant Maximum of * formant Total audio file duration Percentage of voiced parts inside the whole signal Total duration of voiced parts Number of voiced parts Mean duration of voiced parts Standard deviation of voiced parts Minimum duration of voiced parts Maximum duration of voiced parts Total number of pauses in the audio file Mean duration of pauses Standard deviation of pause duration Minimum of pause duration Maximum of pause duration Mean PSD in the frequency range 0–500 Hz Mean PSD in the frequency range 500–1000 Hz Mean PSD in the frequency range 1000–1500 Hz Mean PSD in the frequency range 1500–2000 Hz Mean PSD in the frequency range 2000–2500 Hz Mean PSD in the frequency range 2500–3000 Hz Mean PSD in the frequency range 3000–3500 Hz Mean PSD in the frequency range 3500–4000 Hz Mean PSD in the frequency range 4000–4500 Hz Mean PSD in the frequency range 4500–5000 Hz Mean PSD in the frequency range 5000–5500 Hz

* stands for 1, 2, and 3. These parameters were computed for the first, second, and third formant (respectively, F1, F2, and F3).

**Table 2:** Perceptual indices conversion for multiclass classification experiments.

Values of Perceptual Indices	Class	Class Values
**[0–3]**	Severe	1
**[4–6]**	Moderate	2
**[7–10]**	Mild	3

**Table 3:** Patients’ distribution considering the G index as a grouping variable.

Class	Number of Patients
**Severe**	13
**Moderate**	13
**Mild**	2

**Table 4:** Best classification results for the first AI experiment.

Response Variable	LOSO Cross—Validation Accuracy	Model	Hyperparameters
**G**	82%	KNN	K = 2; d = spearman; w = inverse
**R**	86%	KNN	K = 2; d = spearman; w = equal
**B**	79%	KNN	K = 7; d = cityblock; w = squaredinverse
**Intelligibility**	54%	KNN	K = 17; d = seuclidean; w = squaredinverse
**Fluency**	68%	RF	C = twoing; v = 48; l = 2; s = 1; N = 20
**Voicing**	61%	KNN	K = 11; d = spearman; w = equal
**Spasmodicity**	71%	KNN	K = 3; d = seucliean; w = inverse

**Table 5:** Most relevant features obtained by averaging and normalizing LIME predictors’ weights for perceptual indices in the first AI experiment.

Response Variable	Features
**G**	% voiced, F0 mean
**R**	F2 median, % voiced
**B**	F1 median, PSD III
**Intelligibility**	F0 max, T0(F0 max)
**Fluency**	PSD I
**Voicing**	F0 mean, PSD I
**Spasmodicity**	F1 median, PSD VIII

**Table 6:** Most relevant features obtained by averaging and normalizing LIME predictors’ weights for the G index in the second AI experiment.

Response Variable	Features
**G**	Spasmodicity, R, Voicing

**Table 7:** Confusion matrix: evaluation metrics for the second AI experiment.

Parameter	Class 1 (Severe)	Class 2 (Moderate)	Class 3 (Mild)
**Precision**	0.92 (0.75–1.00)	0.86 (0.67–1.00)	1.00 (0.00–1.00)
**Sensitivity**	0.92 (0.73–1.00)	0.92 (0.75–1.00)	0.50 (0.00–1.00)
**Specificity**	0.93 (0.77–1.00)	0.87 (0.67–1.00)	1.00 (0.00–1.00)
**F-score**	0.92 (0.78–1.00)	0.89 (0.74–1.00)	0.67 (0.00–1.00)
**AUC**	0.91 (0.77–1.00)	0.87 (0.71–1.00)	0.69 (0.00–1.00)

**Table 8:** Statistical analysis results of the first AI experiment. The *p*-values are reported in brackets.

**G**	**R**	**Intelligibility**
**F0 mean (0.012)** **F0 median (0.043)** **F0 max (0.003)**	F0 max (0.014) F1 mean (0.037) F2 mean (0.038) F2 median (0.006) % voiced (0.045)	F0 mean (0.041) F0 max (0.008) F1 std (0.045) Pause duration min (0.049)
**Fluency**	**Voicing**	**Spasmodicity**
**NNE (0.037)** **PSD I (0.010)**	F0 mean (0.005) F0 max (0.001) F2 min (0.030)	F1 mean (0.018) F1 median (0.050) F1 min (0.004) F2 mean (0.024) F2 median (0.032) F2 max (0.043) % voiced (0.024) Number of units (0.050) Number of pauses (0.050)

## Data Availability

The data presented in this study are available on request from the corresponding author.
